# Let’s decide what would be convincing, conduct randomized trials with rigorous comparison conditions, and report tests of moderation and publication bias in meta-analyses

**DOI:** 10.1371/journal.pdig.0000127

**Published:** 2022-11-03

**Authors:** Simon B. Goldberg, John Torous, Shufang Sun

**Affiliations:** 1 Department of Counseling Psychology, University of Wisconsin-Madison, Madison, Wisconsin, United States of America; 2 Center for Healthy Minds, University of Wisconsin-Madison, Madison, Wisconsin, United States of America; 3 Division of Digital Psychiatry, Beth Israel Deaconess Medical Center, Harvard Medical School, Boston, Massachusetts, United States of America; 4 Department of Behavioral and Social Sciences, Brown University School of Public Health, Providence, Rhode Island, United States of America; 5 Mindfulness Center, Brown University, Providence, Rhode Island, United States of America; The University of Sydney, AUSTRALIA

## Abstract

We appreciate Jacobson and colleagues’ thoughtful commentary on our meta-review of mobile phone-based interventions for mental health. In this response, we address 2 issues raised: requiring low to moderate heterogeneity (I^2^ < 50%) and requiring no evidence of publication bias for evidence to be classified as “convincing.” While we agree these represent a high bar, we disagree that these requirements are destined to fail. Other effect sizes reported in the literature, including effect sizes related to mental health interventions and effect sizes related to mobile health (mHealth) interventions (although not their combination) have met requirements for convincing evidence. Jacobson and colleagues argue that features of the mHealth interventions may produce heterogeneity when meta-analyses combine across intervention types. However, several of the effect sizes we reviewed were based on relatively homogeneous portions of the literature and many of the effect sizes we reviewed showed low to moderate heterogeneity. Ideally, future meta-analyses will examine intervention features as moderators of treatment effects. While an absence of publication bias may be a stringent criterion, all but 2 of the 34 effect sizes we reviewed did not report formal tests of publication bias. Clearly, there is a need to reach consensus on how the strength of evidence for mHealth interventions can be evaluated. From our perspective, convincing evidence will ultimately come from large-scale randomized controlled trials employing rigorous comparison conditions along with meta-analyses that do not combine across control condition types, that examine theoretically important moderators, and report formal tests of publication bias. It is this kind of evidence that the public, the clinicians, and the scientific community may need to encourage adoption of mHealth interventions for mental health treatment and prevention.

We appreciate Jacobson and colleagues’ thoughtful commentary on our meta-review [1]. They raise many important points, some of which we address here and several of which we believe are crucial, unresolved issues that the field will have to collectively address.

We start by saying we fully agree with Jacobson and colleagues’ enthusiasm about this area. We adopted nomenclature from prior methodological work on umbrella reviews [2] and used the term “convincing” to refer to the highest level of evidence. While we believe the meaning and intent was clear, we suspect that noting in the abstract that the literature included several highly suggestive effects may have resulted in a different media representation. Beyond terminology, Jacobson and colleagues raise 2 specific points regarding our methodology: requiring low to moderate heterogeneity (I^2^<50%) and requiring no evidence of publication bias. Of note, these are not standards that we invented, but have been applied previously for evaluating mental health interventions [3].

We agree with Jacobson and colleagues that these 2 features represent a high bar. However, we disagree that these requirements are destined to fail. There have been previous meta-reviews that have found effect sizes meeting these criteria, along with the additional recommended [2] requirements (*N* > 1,000, *p* < 0.000001) [3]. In our own in preparation work focused on other parts of the mobile health (mHealth) literature, we have found effect sizes that meet requirements for convincing evidence. We think it is likely convincing effects will emerge for mHealth mental health interventions in the coming years.

Jacobson and colleagues note that low to moderate heterogeneity is unlikely to occur when meta-analyses combine heterogeneous mHealth intervention types that vary in theoretically important ways (e.g., intent, features, goals). We agree in principle that heterogeneity may be related to these aspects of the interventions. Meta-analysis can be an ideal method for evaluating this empirically, as these study-level features can be tested as moderators. Unfortunately, only 1 effect size [4] of the 34 we reviewed tested moderators, and none of the features evaluated (including elements of the interventions) predicted treatment effects. Thus, based on the meta-analytic evidence, it is largely unclear whether features of the mHealth interventions themselves predict treatment effects. The thorough testing of moderators within samples of studies that do not combine control condition types (see [1] for a discussion of this issue) is a crucial future direction.

Although it is true the primary studies included in our meta-review were heterogenous, we should clarify that the meta-analyses we reviewed often included effect sizes representing relatively homogeneous portions of the literature, such as meditation apps [5], standalone smartphone apps [6], or text message-based smoking cessation interventions [7] (and, in fact, many of the effect sizes we extracted showed low to moderate heterogeneity). As we reviewed meta-analyses rather than conducting a meta-analysis ourselves in this study, we did not make decisions regarding which interventions should be combined. Precisely which portions of the literature should be combined in meta-analyses is an important issue to resolve, particularly given the wide variety of mHealth approaches that are being tested.

Finally, we would like to clarify the issue of requiring an absence of publication bias. Fusar-Poli and colleagues [2] note 2 forms of potential bias that must be absent for convincing evidence: small-study effects and excess significance bias. In our review, we only evaluated the first of these (small-study effects) which, following Dragioti and colleagues [3], we operationalized as an absence of publication bias based on tests of funnel plot asymmetry (i.e., Egger’s regression). Unfortunately, only 2 of the 34 effect sizes evaluated formally tested for publication bias. We hope that future meta-analyses formally test for publication bias within portions of the literature that do not combine control condition types.

Ultimately, we believe that convincing evidence for mHealth interventions for mental health is out there to be found and it is incumbent upon mHealth researchers to conduct rigorous investigations to find it [8]. Convincing evidence will likely come from large-scale randomized controlled trials, particularly trials using rigorous comparison conditions. Future meta-analyses, sensitive to the nature of control conditions and aspects of the interventions being tested, will be essential for, one might say, convincing the public, the clinicians, and the scientific community to adopt mHealth interventions as a part of mental health treatment and prevention.
